# Spectroscopic and Computational Characterization of the Adsorption of Quaternary Ammonium Ions on Ceria-Supported Colloidal Silica Particle Films

**DOI:** 10.1021/acs.langmuir.5c01955

**Published:** 2025-08-13

**Authors:** Nicole M. Smiddy, Jamie L. Sedlacko, Charles M. Ramia, Patrick E. Schneider, Dean J. Campbell, Wayne B. Bosma, Edward E. Remsen

**Affiliations:** Mund-Lagowski Department of Chemistry and Biochemistry, 6496 Bradley University, 1501 West Bradley Avenue, Peoria, Illinois 61625, United States

## Abstract

The adsorption of molecular ions from aqueous solutions onto colloidal silica is utilized across a wide range of technologies. Measurement of such interactions using attenuated total reflectance/Fourier transform infrared (ATR/FT-IR) spectroscopy presents challenges due to the instability of colloidal silica particle films on an ATR’s diamond crystal internal reflection element (IRE). Here, the instability of silica is addressed by first depositing a thin film of colloidal ceria particles on the IRE to improve silica’s adhesion. This enables the application of ATR/FT-IR spectroscopy to characterize the adsorption of quaternary ammonium ions, including the poly­(2-(trimethylamino)­ethyl methacrylate) (*p*TMAEM) and compounds modeling *p*TMAEM’s side chain functional groups, tetramethylammonium (TMA) and acetylcholine (AChl) ions, on low- and high-silanol density silica films at varying pH values. Density functional theory (DFT) calculations are performed to model the TMA–silica and *p*TMAEM–silica complexes and predict their infrared (IR) spectra. The adsorption of TMA, AChl, and *p*TMAEM to silica is demonstrated by IR bands in the range of 950–1250 cm^–1^, which were dominated by transverse optical and longitudinal optical phonon modes. Langmuir adsorption constants based on optical phonon modes indicate that TMA is more strongly adsorbed to the low-silanol density films, whereas *p*TMAEM shows a stronger adsorption to silica overall, regardless of the silanol density. This suggested that both electrostatic and nonelectrostatic interactions contribute to TMA’s adsorption, while the polymeric nature of *p*TMAEM enabled adsorption due to strong electrostatic interactions via multiple adsorption events. AChl’s relatively weak adsorption to silica prevented the calculation of a Langmuir adsorption constant; however, the IR spectra suggested any adsorption was primarily due to the quaternary ammonium ion. The application of ATR/FT-IR spectroscopy in combination with DFT allowed a complete characterization of the adsorption of the quaternary ammonium ion onto ceria-supported colloidal silica, which may also be applied to other molecular ions, further characterizing and optimizing technologies employing similar materials.

## Graphical abstract

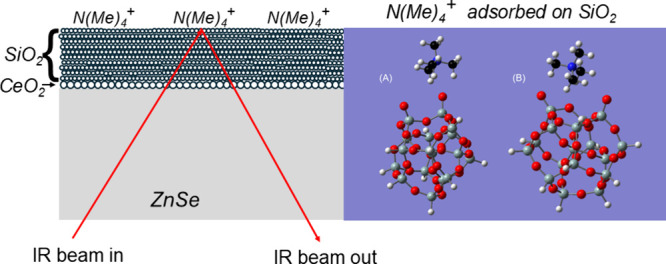

## Introduction

Colloidal silica is a well-established and vital material in a host of important technological applications, including drug delivery, medical diagnostics, antibiotics, eco-friendly pesticides, energy storage, catalysts, environmental remediation, and polishing abrasives for integrated circuit fabrication.^1–4^ The versatility of colloidal silica in enabling this wide range of applications stems from the reactivity of its chemically distinct surface silanol and siloxane groups in water,^5^ which facilitate surface functionalization chemistries and adsorption behaviors. A representative example of the latter property is the adsorption of organic quaternary ammonium ions such as tetramethylammonium (TMA) ions,^6,7^ cationic surfactants,^8^ and polyelectrolytes^9^ from aqueous solution. Attractive electrostatic interactions between positively charged quaternary ammonium ions and negatively charged, deprotonated silanol surface groups mainly account for the adsorption of these species on colloidal silica. However, nonelectrostatic interactions, such as hydrophobic and dispersion interactions,^9^ have also been identified as promoting adsorption on colloidal silica surfaces. While previous reports offer insight into quaternary ammonium ion adsorption on colloidal silica, details of these adsorptive interactions at the molecular level remain to be fully elucidated, such as the energetically preferred molecular orientations of the adsorbed ions. In this work, a combination of experimental and computational methods, attenuated total reflectance/Fourier transform infrared (ATR/FT-IR) spectroscopy, and density functional theory (DFT) calculations were applied to characterize the molecular adsorption of quaternary ammonium ions onto colloidal silica films under aqueous conditions. Specifically, ATR/FT-IR spectra were collected for the poly­(2-(trimethylamino)­ethyl methacrylate) (*p*TMAEM) ion and ions modeling the polycation side chain functional groups, TMA and acetylcholine (AChl), on colloidal silica particle films prepared with low- and high-silanol density silicas. From the acquired spectra, Langmuir adsorption constants can be calculated based on optical phonon modes. This spectroscopic approach has been reported previously^10–16^ for characterizing molecule adsorption on a variety of thin metal oxide particle films, including silica particle films.^13–15^ However, the collection of ATR/FT-IR spectra for molecules on silica films presents known challenges due to silica’s instability on a diamond crystal internal reflection element (IRE) under aqueous conditions.^13^ Therefore, a distinguishing feature of the ATR/FT-IR studies reported here is the optimization and application of a ceria-supported silica film, which is composed of a thin lower layer of colloidal ceria particles to stabilize the upper film of colloidal silica.

In combination with the experimental ATR/FT-IR spectroscopy results, computational modeling of TMA ion adsorptive interactions with the silica surface was performed by using density functional theory (DFT). DFT has been used previously to model vibrations in silica,^17,18^ providing good qualitative agreement with experimental IR spectra. In addition, DFT of species adsorbed to silica surfaces has provided binding geometries, as well as adsorption energies^19–21^ and IR signatures of different binding modalities.^20,21^ Here, the complementary application of experimental and computational techniques has enabled an improved molecular understanding of the adsorption behaviors of quaternary ammonium ions on colloidal silica surfaces under aqueous conditions.

## Experimental and Computational Methods

### Materials

Dispersions of two sol–gel colloidal silicas, one with a silanol density of 2.6 OH/nm^2^ and the other with a silanol density of 1.6 OH/nm^2^, were purchased from Fuso Chemical Co., Ltd. (Osaka, Japan). The higher-silanol density silica (SiOH_high_) and the lower-silanol density silica (SiOH_low_) were received as 37% (w/v, pH 7.3) and 89% (w/v, pH 6.5) aqueous dispersions, respectively. Particle size distributions for the dispersions were determined by centrifugal disc sedimentation,^22^ which yielded the mean weight-average hydrodynamic diameter, *D*_w_, and mean number-average hydrodynamic diameter, *D*_n_, for the dispersions. Colloidal ceria (CeO_2_) was purchased from Advanced Nano Products Co., Ltd. (Sejong, Korea), as a 6.25% (w/v, pH 3.3) aqueous dispersion that had a *D*_w_ of 113 nm determined previously.^16^ The values of *D*_w_ determined by centrifugal disc centrifugation for SiOH_low_ and SiOH_high_ were 42 and 48 nm, respectively. Dilutions of the as-received particle dispersions were made with pH-adjusted, doubly deionized (18 MΩ cm) water.

Poly­(2-(trimethylamino)­ethyl methacrylate) chloride was purchased from AkzoNobel, N.V. (Amsterdam, The Netherlands). Chloride salts of tetramethylammonium and acetylcholine were purchased from Sigma-Aldrich, Inc. (St. Louis, MO) and were >95% pure. All other chemicals used in this study were reagent grade or higher in purity. Aqueous stock solutions of the chloride salts of TMA, AChl, and *p*TMAEM were prepared by dilution with doubly deionized water followed by adjustment of the solution pH using either sodium hydroxide or nitric acid. The pH of all solutions was determined using a model FE20 pH meter (Mettler Toledo Inc., Columbus, OH) operated with a calibrated glass pH electrode (Cole-Parmer Instrument Co., Vernon Hills, IL).

### Preparation of Ceria-Supported Silica Films

The general procedure used to prepare ceria-supported silica films on the diamond IRE was a modification of the procedure described previously^16^ for the preparation of pure ceria films on the IRE. One microliter of a 0.68 mg/mL, pH 4 ceria dispersion was deposited as a drop on the diamond surface and air-dried for 10 min to allow the water to evaporate and form a thin ceria film with the minimal thickness (ca. 0.23 μm) required to stabilize a thicker silica film deposited on ceria film’s surface. Once the film was formed, 1 μL of a 4.8 mg/mL dispersion of either SiOH_high_ or SiOH_low_ adjusted to pH 6 was deposited on the ceria film layer. The silica films were not taken to complete dryness, as this led to delamination of the silica film under water flow through the ATR flow cell. A drying time of approximately 7 min was sufficient to form a stable silica film with an estimated thickness of ca. 1.5 μm. The resulting ceria-supported silica films were then conditioned by flowing a 10 mM NaCl, pH 6 solution over the films for 10 min to stabilize the electrical double layer of the films prior to the collection of IR spectra.

### ATR/FT-IR Spectroscopy

ATR/FT-IR spectra were recorded over the range of 4000–400 cm^–1^ with a 4 cm^–1^ resolution using a Thermo-Scientific NEXUS 470 FT-IR spectrometer equipped with a flow-through sample cell, a deuterated triglycine sulfate detector operated at room temperature, and a Smart Endurance ATR accessory employing a 42°, single-reflection, 0.75 mm diameter, diamond ATR crystal internal reflection element (IRE) with ZnSe focusing optics. Data analysis of recorded spectra followed procedures previously described in detail.^16^ In summary, a spectrum of the ceria-supported silica film equilibrated in doubly deionized water and adjusted to pH 6 was recorded as the background absorption spectrum. The background spectrum was subtracted from IR absorbance spectra of the quaternary ammonium ion solutions in equilibrium with the films to provide the IR spectrum of adsorbed ions on the films and nonadsorbed ions in solution. Following the recording of IR spectra, with continuous flushing of the films with pH 6, doubly deionized water was used to desorb the ions and restore the background absorption spectrum.

Peak frequencies for adsorbed quaternary ammonium ions producing broad absorbance bands were determined based on the wavenumber corresponding to the band center. Frequencies for shoulders on absorbance bands were evaluated from the first- and second-derivative spectra. Corresponding peak absorbances were determined for spectra fitted over the desired wavenumber range with a linear baseline.

### Adsorption Isotherms

The analysis of Langmuir adsorption isotherms and the determination of the Langmuir constant, *K*, for TMA and *p*TMAEM adsorption on SiOH_high_ and SiOH_low_ films employed the ATR/FT-IR methodology described previously.^16^ *K* from adsorption isotherms was calculated by the linear regression analysis of eq 1.

1 $$\frac{c}{A}=\frac{c}{A_{\max}}+\frac{1}{KA_{\max}}$$

where *c* is the concentration of the absorbate (TMA or *p*TMAEM), *A* is the corresponding absorbance, and *A*_max_ is the maximum absorbance. *K* was determined from the *y*-intercept of the linear regression fitting (1/*KA*_max_) after substitution of *A*_max_ determined from the slope of the fitting (1/*A*_max_). Calculations for the analysis of *K* were performed with ORIGIN version 2024b (OriginLab Inc., Northampton, MA).

### Scanning Electron Microscopy-Energy Dispersive X-ray Analysis (SEM-EDX)

SEM-EDX data were acquired by using an FEI model XL30 scanning electron microscope. Test films on a silicon wafer were prepared from the same silica and ceria dispersions used to form ceria-supported silica films on the diamond IRE. Prior to test film deposition, the wafer was scored on the opposite face from where the film was to be placed. Following deposition and drying, the test film was fractured by applying a quick downward force at the score mark. The fracture produced two samples, each with one edge of the cross section exposed. The film was then sputter coated with a Au/Pd alloy (60:40 weight ratio, ∼5 nm thick) to image its cross section and determine the chemical composition of the film by EDX as a function of the height above the wafer’s surface.

### Density Functional Theory (DFT) Calculations

Infrared spectra of silica and silica–TMA complexes were calculated using the Gaussian-16 software package,^23^ at the B3LYP/6-31+G* level of theory, using the C-PCM continuum solvation model.^24^ The silica surface was modeled using small clusters, where an average spectrum was taken using 10 different clusters, each with the formula [Si_17_O_30_H_10_]^2–^. The overall structures of the clusters differ in the locations of two external silanolate oxygen atoms, each of which has a formal charge of −1. Different clusters were made by interchanging hydrogen atoms and silanolate oxygen atoms on the surface of the cluster. In some clusters, the two silanolate oxygen atoms were vicinal to each other (i.e., bonded to adjacent silicon atoms with a bridging oxygen atom); in others, the silanolate oxygen atoms were more distant from each other. TMA–silica complexes were formed by performing full geometry optimizations with the TMA ion located near one or both silanolate oxygen atoms.

Infrared spectra for the silica clusters and the silica–TMA complexes were calculated using the normal-mode frequencies and IR intensities calculated by Gaussian and convoluting with a Gaussian function of 20 cm^–1^ at half-maximum. The IR difference spectrum was calculated using an average of spectra for 24 different TMA–silica complexes, based on a total of 10 different silica clusters. The spectra were averaged using Boltzmann weighting factors calculated from the binding energies of the individual complexes. Then, the average IR spectrum for the silica–TMA complexes was subtracted from the average IR spectrum for the 10 silica structures. Infrared frequencies for the calculated spectra were not scaled.

## Results and Discussion

### Ceria-Supported Silica Films

Previous studies have described the deposition of silica films on an ATR’s IRE to measure molecular adsorption on silica in an aqueous solution.^13–15^ For example, the adsorption of a silica film on a diamond IRE was reported^13^ to be facilitated by the deposition of silica at pH 2.5, which generally enabled the IR analysis of a surfactant’s adsorption on silica; however, the pH-stabilized film deteriorated at high surfactant concentrations. Initial attempts in the present study to deposit a pure silica thin film prepared from the SiOH_high_ and SiOH_low_ dispersions at pH 2.5 on the diamond surface of the IRE did not produce stable films. Deposited films delaminated nearly immediately from the IRE’s surface under water flow through the ATR’s flow cell accessory. This problem was eliminated by first depositing a thin ceria film on the IRE’s surface followed by deposition of a thick silica film on the partially dried ceria film. The attractive electrostatic interaction at the interface between the silica and ceria particle films between pH 4 and 8 ^25,26^ stabilized the adhesion of the silica film.

Prior to collection of IR spectra, SEM-EDX analyses of the cross section of a test ceria-supported SiOH_high_ film deposited on a silicon wafer confirmed the applicability of the ceria-supported silica film preparation procedure. As shown in Figure 1, the EDX profiles of a representative film’s cross section demonstrated a 1.5 μm top layer of SiOH_high_ (green trace for the Kα1 line for silicon) and an underlying 1.5 μm layer of ceria particles (blue trace for the Mα line of cerium) adhering to the silicon wafer’s surface. As expected, oxygen was detected in the silica and ceria films using the EDX profile for the oxygen content (red trace for the Kα1 line of oxygen).

**Figure 1:**
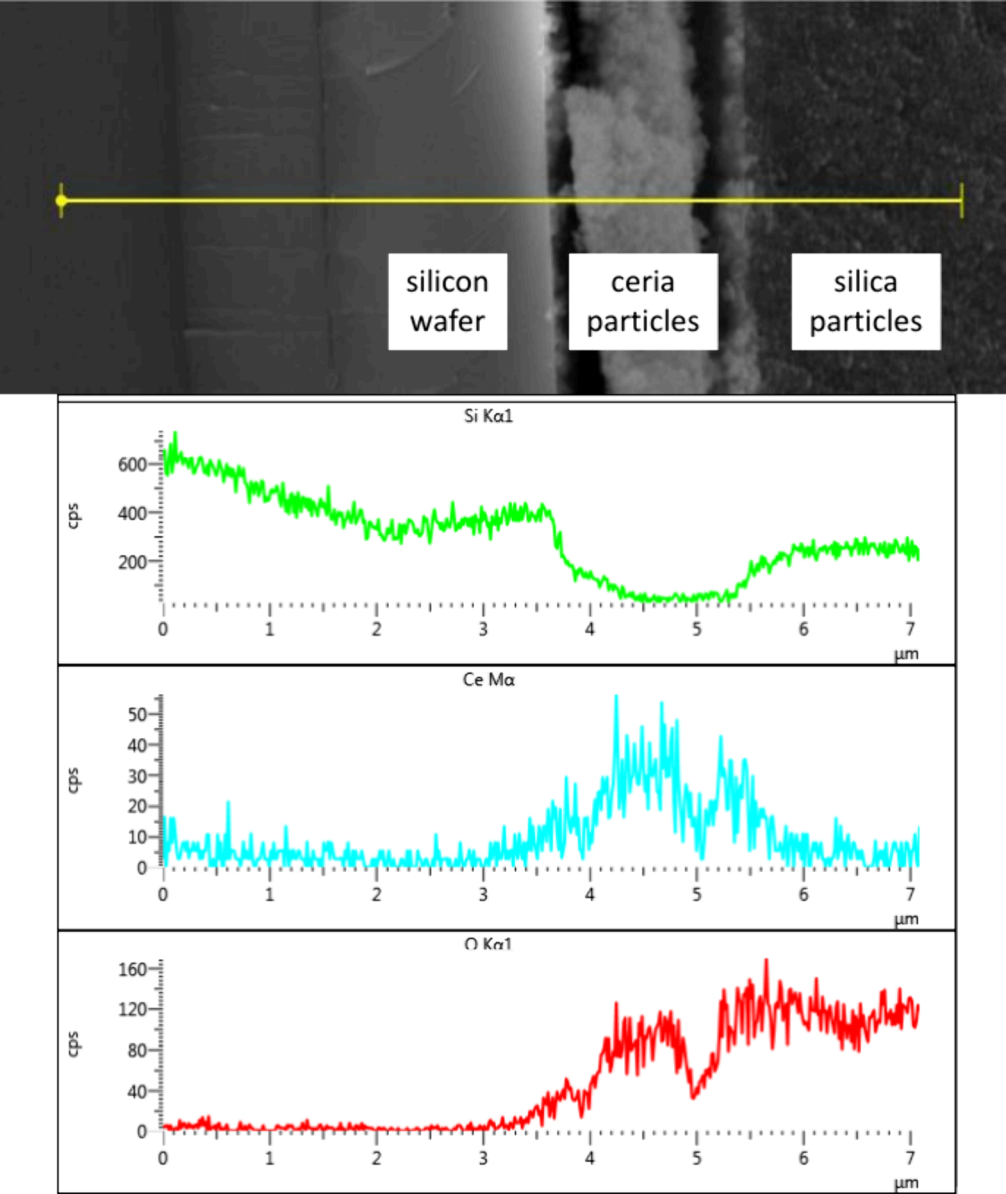
SEM image (top) and EDX profiles (bottom) of a cross-sectioned, ceria-supported SiOH_high_ test film. The green profile corresponds to the silicon Kα1 line response for the silica particle film, the blue profile the cerium Mα line response for the ceria particle film, and the red profile the oxygen Kα1 line response across both particle films.

Under the deposition conditions employed, evidence of interpenetration of silica and ceria layers was noted from the Si Kα1 and Ce Mα signals at the interface defined by the 5.3–5.7 μm region of the EDX profiles in Figure 1. This finding suggested that an interfacial layer formed between the deposited ceria and silica films on the IRE surface could potentially contribute to ATR/FT-IR spectra of adsorbed ammonium ions on a ceria-supported silica film.

### IR Spectroscopy and Stability of Ceria-Supported Silica Films

ATR/FT-IR spectra recorded between 900 and 1400 cm^–1^ were used to assess the stability of the films in contact with both acidic and basic solutions. Representative results obtained for pH 4 and 10 water flowing over the surface of a ceria-supported SiOH_high_ film with solution exposure times ranging from 1 to 36 min are shown in Figure 2. Significant differences between the film spectra were observed as a function of solution pH.

**Figure 2:**
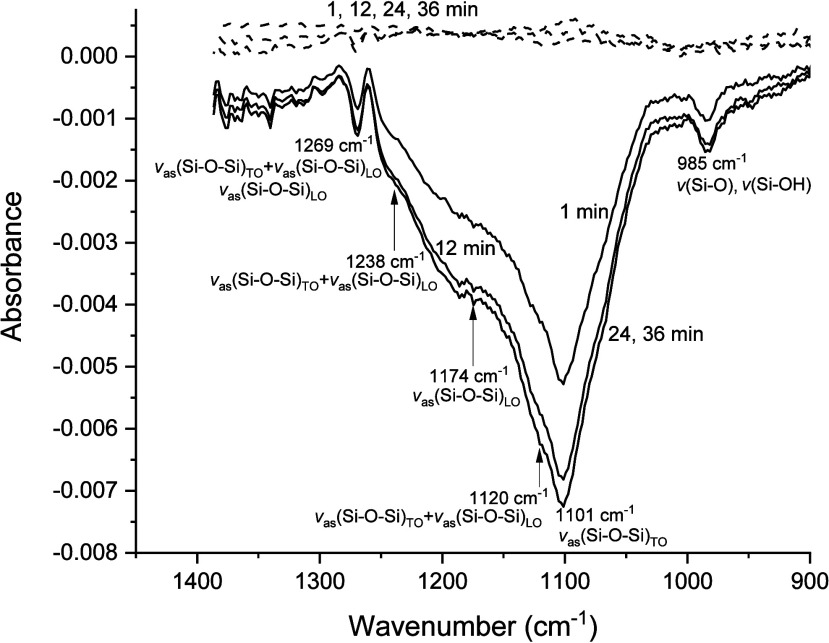
Representative IR spectra of a ceria-supported SiOH_high_ film as a function of pH and exposure time (1, 12, 24, and 36 min). Dashed lines correspond to pH 4 spectra, and solid lines correspond to pH 10 spectra.

Negative absorbance peaks (minima) obtained for the pH 10 solutions were the product of progressive dissociation of the silica film from the ceria-coated ATR’s diamond IRE with increasing exposure time at pH 10. The decreasing thickness of the deposited silica film produced IR absorbance minima relative to the initial background spectrum (time = 0 min) of the film. Smaller relative decreases in absorbance after 24 min of pH 10 exposure suggested nearly complete dissociation of the silica films at longer equilibration times.

Three negative IR absorbance peaks between 900 and 1400 cm^–1^ are apparent in Figure 2 with the indicated frequencies at 985, 1101, and 1269 cm^–1^ corresponding to the absorbance minima of the peaks. Additional minima in the spectra are evidenced by shoulders on the broad negative absorbance band. Frequencies corresponding to the minima for the peaks and shoulders were determined from spectra collected after exposure for 36 min to the pH 10 solution. Resulting peak frequencies of 1120, 1174, and 1238 cm^–1^ for the shoulders are indicated by arrows in Figure 2.

The assignment of the IR spectrum of silica in the range of 900–1400 cm^–1^ is well established,^27–31^ and asymmetric Si–O–Si stretching modes, ν_as_(Si–O–Si), dominate this range. Published assignments suggested that the main peak at 1101 cm^–1^ was a transverse optical (TO) phonon, ν_as_(Si–O–Si)_TO_. TO phonons in silica films generally show stronger IR absorbance than LO phonons^30,31^ except when an oblique IR beam angle of incidence is used^30^ as in the present study that employed a 42° angle of incidence. The shoulder at 1178 cm^–1^ was assigned to a longitudinal (LO) phonon, ν_as_(Si–O–Si)_LO_, while the shoulders at 1120 and 1238 cm^–1^ corresponded to mixed TO–LO phonons, ν_as_(Si–O–Si)_TO–LO_. The peak at 1269 cm^–1^ was assigned to overlapping mixed TO–LO and LO phonons. The peak observed at 985 cm^–1^ was consistent with both ν­(Si–O) and ν­(Si–OH) stretching modes.^31^

Spectra recorded at pH 4 (dashed lines in Figure 2) exhibited no detectable changes between 1 and 30 min, indicating no detectable silica film loss over this time due to dissociation into flowing water at pH 4. The quantitative pH dependence of film stability for both SiOH_high_ and SiOH_low_ films was evaluated between pH 2 and 10. Absolute values of the integrated area of the band corresponding to the 1101 cm^–1^ IR absorbance minimum after exposure to flowing water for 30 min were determined as a function of pH. Results for a set of SiOH_high_ films, presented in Figure 3, indicate that silica film loss was negligible between pH 4 and 8. A similar trend was found for the SiOH_low_ film except that minimal silica film loss was only observed between pH 4 and 6 (Figure S1). Therefore, all IR spectra of the quaternary ammonium ion solutions collected in the presence of both SiOH_high_ and SiOH_low_ films employed pH 6 solutions. Since pH 6 is notably higher than the isoelectric point of colloidal silica (pH ∼2),^32^ the silica film surfaces had a net negative charge during the collection of IR spectra for the quaternary ammonium ion solutions equilibrated with the films.

**Figure 3:**
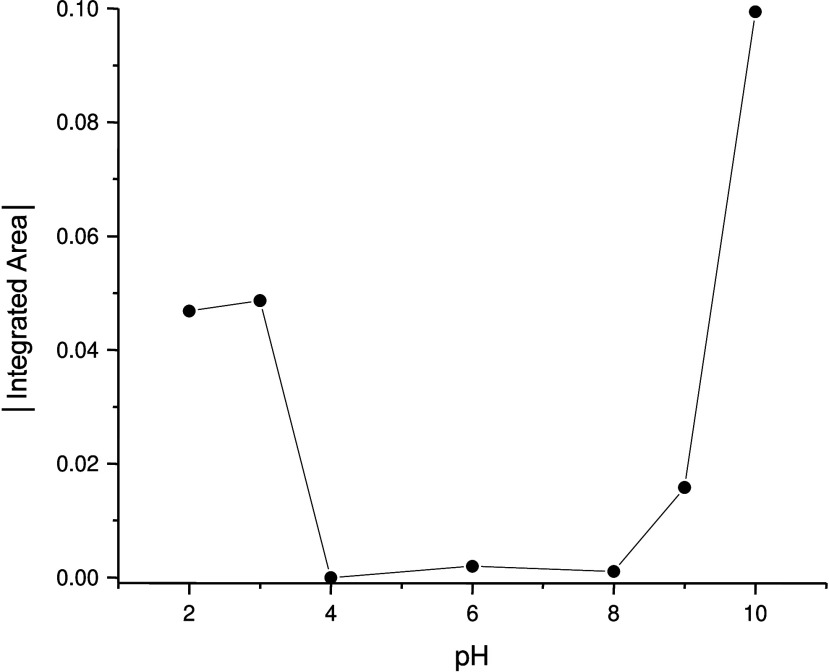
Absolute value of the integrated area of the band corresponding to the 1101 cm^–1^ IR absorbance minimum in the ATR/FT-IR spectra for ceria-supported silica films prepared with SiOH_high_ after exposure to flowing water for 30 min with the pH ranging from 2 to 10.

### Infrared Spectroscopy of Adsorbed TMA Ions

Numerous investigations using a wide variety of analysis methods^33–37^ have demonstrated that the adsorption of compounds containing positively charged ions on silica occurs mainly through electrostatic interactions with the film’s surface oxygen atoms from siloxane (Si–O–Si) groups and silanolates (Si–O^–^) produced by deprotonation of isolated, geminal, vicinal, and terminal silanol groups. The chemical structures of the quaternary ammonium ions used in the present study shown in Figure 4 suggested that attractive electrostatic interactions with silica film surfaces would produce the adsorption of the ions. ATR/FT-IR spectra between 900 and 1500 cm^–1^ of pH 6 solutions of the quaternary ammonium ions, shown in Figure 5, were recorded in the presence and absence of SiOH_low_ and SiOH_high_ films. Prior to spectral analysis of the interactions between a silica film’s surface and quaternary ammonium ions, the film was equilibrated for 5 min with the flowing quaternary ammonium ion solution to ensure that adsorption of the ions on the films reached equilibrium. The 5 min equilibration time was based on the comparison of IR spectra recorded sequentially for a TMA, pH 6 solution in contact with a silica film. This analysis showed no changes in the IR spectrum of the adsorbed TMA ion after equilibration with the TMA solution for 5 min.

**Figure 4:**
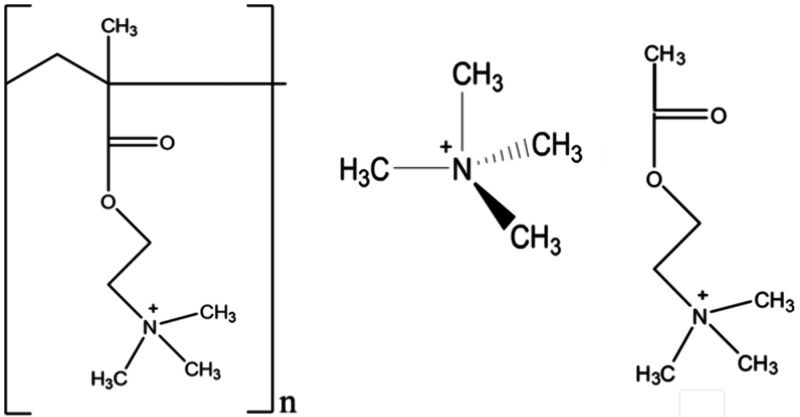
Chemical structures of the TMA and AChl cations, and TMAEM, the repeating unit cation of *p*TMAEM.

**Figure 5:**
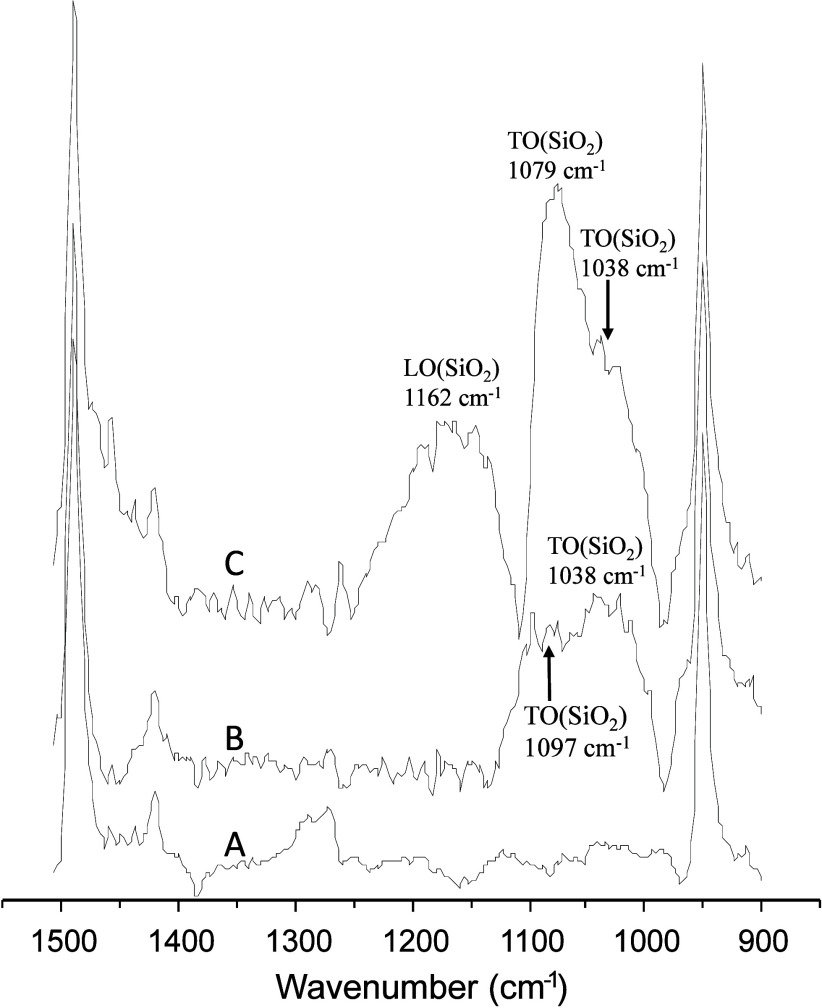
Overlay of stacked IR spectra for pH 6 solutions of (A) 40 mM TMA, (B) 40 mM TMA with a ceria-supported SiOH_low_ film, and (C) 40 mM TMA with a ceria-supported SiOH_high_ film.

The spectrum of 40 mM TMA in the presence of the SiOH_high_ film (Figure 5, spectrum C) exhibited a broad absorbance peak at 1079 cm^–1^ with a shoulder at 1038 cm^–1^, and an additional broad absorbance at 1162 cm^–1^. The spectrum of 40 mM TMA in the presence of the SiOH_low_ film (Figure 5, spectrum B) exhibited a pair of broad, overlapping absorbances at 1097 and 1038 cm^–1^. Based on the assignments presented in Figure 2, the 1079 and 1038 cm^–1^ peaks of the SiOH_high_ film (Figure 5, spectrum C) and the absorbances at 1097 and 1038 cm^–1^ of the SiOH_low_ film (Figure 5, spectrum B) were assigned to TO phonons, TO­(SiO_2_). Similarly, the broad absorbance at 1162 cm^–1^ of the SiOH_high_ film (Figure 5, spectrum C) was assigned to LO phonons, LO­(SiO_2_). The detection of LO phonons has been interpreted as being indicative of increased porosity in sol–gel-derived silica films.^30^ This finding suggested that the SiOH_high_ film had a lower porosity than the SiOH_low_ film due to the lack of absorbance in the range of 1200–1250 cm^–1^ of the SiOH_high_ film (Figure 5, spectrum B). The application of LO and TO phonon modes associated with ν_as_(Si–O–Si) for the detection of adsorbed biological molecules on silica nanoparticle and nanorod films incorporated in sandwich-structured immunoassays has been described previously.^38,39^ Comparison of TO- and LO-mode frequencies for the silica films in the presence of TMA (Figure 5) and in the absence of TMA (Figure 2) indicated wavenumber shifts attributable to the formation of TMA–silica complexes at the film surfaces. Previous studies have demonstrated the sensitivity of silica TO and LO frequencies to cation adsorption on colloidal silica particles in aqueous solution, such as shifts in TO frequencies due to the adsorption of potassium ions on silica gel in an aqueous solution.^40^

The potential contribution of the underlying ceria film to the IR spectrum of adsorbed TMA ions was evaluated using relatively thick films (ca. 1.5 μm) of colloidal ceria particles deposited on the diamond IRE as described previously.^16^ After equilibration of the ceria film with pH 6 TMA solutions, no new peaks or other spectral changes were found in the IR spectrum. Adsorption of TMA ions on the ceria species of ceria-supported silica films, therefore, was ruled out as a source of the IR absorbance for adsorbed TMA. This result is consistent with the expected lack of electrostatic stabilization of an adsorbed TMA ion at pH 6 on colloidal ceria particles that would be positively charged due to protonation below colloidal ceria’s isoelectric point (pI = 6.5–7.8).^41,42^

### DFT Calculations

In conjunction with the ATR/FT-IR analysis of TMA adsorption, DFT calculations were used to structurally define TMA–silica complexes formed at the silica film surface due to TMA adsorption and to calculate their corresponding infrared absorption spectra. Figure 6 shows two of the 24 optimized complexes that were used in the computations. Complex A illustrates a TMA ion singly coordinated to the silica complex, while complex B displays a TMA ion in bifurcated coordination. During the calculations, most of the structures that started with the TMA ion in a bifurcated position between two vicinal silanolate atoms converged to a geometry with the TMA associated with only one of the two oxygen atoms; only four of the evaluated structures converged to bifurcated geometries.

**Figure 6:**
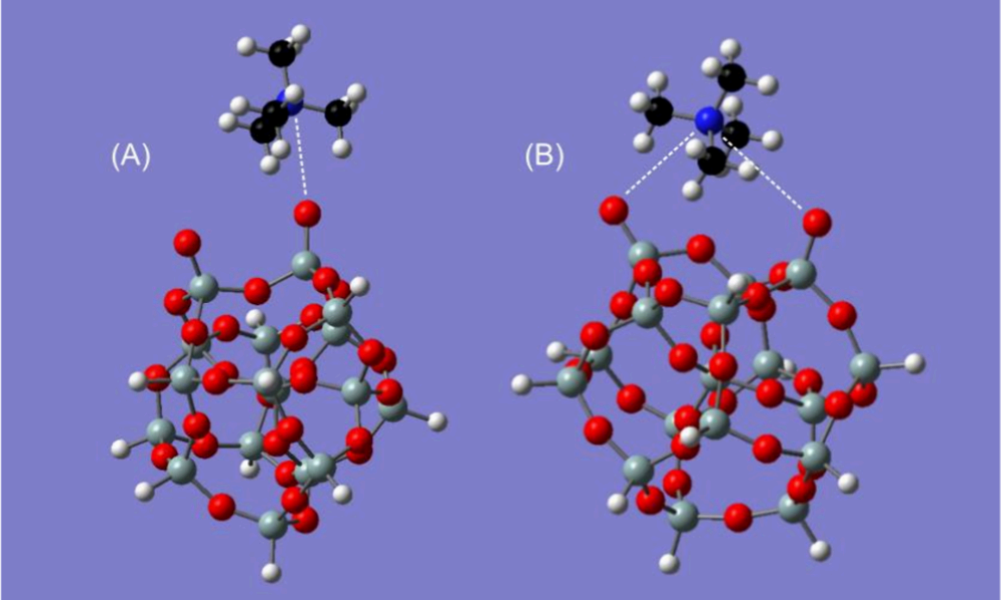
Two of the TMA–silica complexes used in DFT calculations. (A) TMA ion singly complexed with the silica cluster. (B) TMA ion in a bifurcated complex with the silica cluster. Color coding for atoms: red for oxygen, gray for silicon, blue for nitrogen, black for carbon, and white for hydrogen. Dashed lines indicate electrostatic interactions between TMA cations and negatively charged silanolate groups (Si–O^–^) of the silica clusters.

All 24 different complexes studied were very similar in overall stability with binding energies ranging from 2.2 to 3.5 kcal/mol. The four complexes with the TMA ion positioned in bifurcated geometries between two silanolate oxygen atoms were the most weakly bound, at 2.2–2.4 kcal/mol. For the nonbifurcated complexes, the nitrogen–oxygen distances were all between 3.43 and 3.47 Å. For the bifurcated complexes, the distance from the TMA nitrogen atom to the closest silanolate oxygen atom ranged from 3.72 to 4.08 Å. The positioning of the TMA ion in the nonbifurcated complexes was generally similar to that of complex A with Si–O–N angles ranging from 150° to 180°.

The averaged, calculated IR difference spectrum for the TMA–silica complexes is compared to the experimental IR spectrum of 40 mM TMA with a ceria-supported SiOH_high_ film in Figure 7. The calculated spectrum reproduces the experimental spectrum reasonably for the TMA peaks in the range of 1000–1200 cm^–1^. However, peaks appearing at 958 and 1548 cm^–1^ in the computed spectrum had a relative absorbance lower than that in the experimental spectrum. This difference is likely due to the presence of free TMA in solution contributing to the experimental spectrum. Experimental and calculated frequencies in Figure 7 are summarized in Table S1.

**Figure 7:**
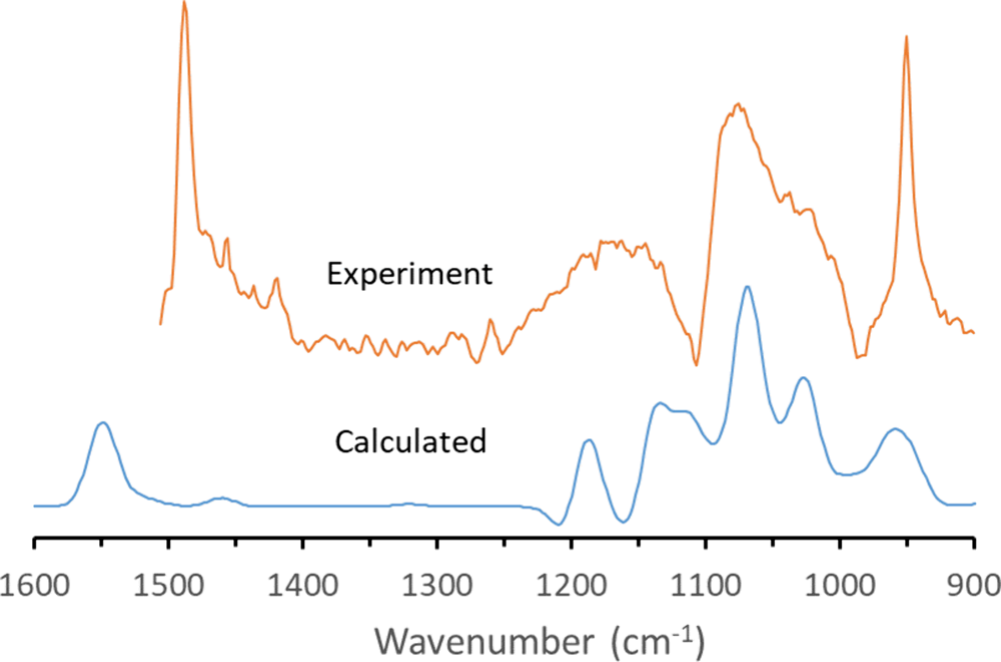
DFT-calculated IR spectrum compared to the experimental spectrum of 40 mM TMA with a ceria-supported SiOH_high_ film.

In the calculated spectrum, there are a total of 55 vibrational modes observed from 900 to 1600 cm^–1^. The calculated normal modes for the TMA–silica complex were animated, so that TMA and silica’s relative contributions to the vibrations could be evaluated. The 958 cm^–1^ peak in the calculated spectrum has contributions from a total of eight normal modes, including two that are predominantly N–C stretching vibrations in TMA, four that are predominantly Si–O–Si stretching vibrations in silica, and two that show significant vibrations in both TMA and silica. The presence of silica-only vibrations, likely shifted relative to the experimental silica vibration frequencies, may explain why the 958 cm^–1^ peak is broader in the calculated spectrum than in the experimental spectrum. One notable difference between the calculated and experimental spectra is the location of the TMA peak at 1489 cm^–1^, which appears at a frequency ∼60 cm^–1^ higher (1548 cm^–1^) than that in the calculated spectrum.

Calculations showed no noticeable contribution from silica’s normal modes between 1200 and 1600 cm^–1^. Instead, there are 17 TMA-dominated vibrational modes in this region, including three CH_3_ bends that contribute to the peak at 1548 cm^–1^ and two CH_3_ bends that produce the weakly absorbing peak at 1460 cm^–1^. The other vibrational modes in that region are too low in absorbance to be visible in the calculated difference spectrum.

The region between 1000 and 1200 cm^–1^ arises from a total of 25 normal modes, of which 18 are predominantly Si–O–Si asymmetric stretching vibrations in silica. Four of the modes have significant vibrational motion both in the silica and in the TMA, while three others, all near 1100 cm^–1^, are almost exclusively asymmetric Si–O–Si stretching vibrations (ν_as_(Si–O–Si)). The TMA-dominated normal modes are much weaker than the surrounding silica and silica–TMA vibrations, with amplitudes of less than 1% of the tallest silica-dominated peaks in that region. The silica vibrations in this region are best described as predominately ν_as_(Si–O–Si), which is consistent with assignments made by previous authors.^30,31^

### Infrared Spectroscopy of Adsorbed *p*TMAEM and AChl Ions

IR spectra in Figures 8 and 9 for 10 mM *p*TMAEM in contact with the SiOH_high_ and SiOH_low_ films, respectively, are comparable to the spectra obtained for TMA ions contacting the films (Figure 5). The baseline-corrected, scaled difference spectra (spectrum C, filled circles) in Figures 8 and 9 revealed absorbance peaks between 985 and 1122 cm^–1^ characteristic of the TO phonon mode of the silica film. One variation noted between the difference spectra was the location of the TO peak maxima. The difference spectrum for the SiOH_high_ film had an absorbance maximum at 1089 cm^–1^, while the corresponding absorbance for the SiOH_low_ film occurred at 1103 cm^–1^. This difference in TO peak maxima was consistent with the results for TMA (Figure 5) in which the TO peak maximum for the SiOH_high_ film (1079 cm^–1^) was at a lower frequency than the SiOH_low_ film (1097 cm^–1^). These findings indicate that the difference spectra in Figures 8 and 9 are consistent with adsorption of *p*TMAEM to form silica–*p*TMAEM complexes.

**Figure 8:**
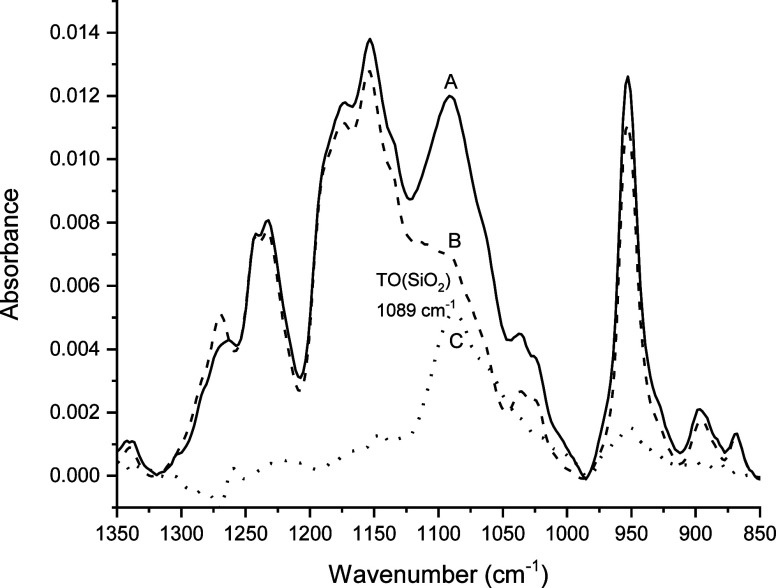
Overlay of IR spectra of pH 6 solutions of 10 mM *p*TMAEM with a SiOH_high_ film (spectrum A, solid line), 10 mM *p*TMAEM with no film (spectrum B, dashed line), and the baseline-corrected, scaled difference spectrum (spectrum C, filled circles = solid line – dashed line).

**Figure 9:**
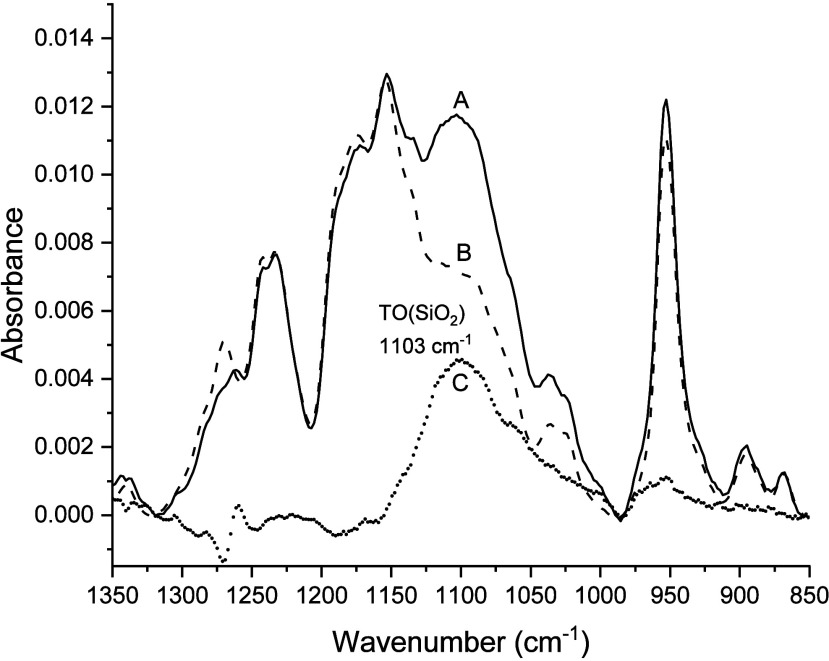
Overlay of IR spectra of pH 6 solutions of 10 mM *p*TMAEM with a SiOH_low_ film (spectrum A, solid line), 10 mM *p*TMAEM with no film (spectrum B, dashed line), and the baseline-corrected, scaled difference spectrum (spectrum C, filled circles = solid line – dashed line).

The potential contribution of the side chain functional groups of *p*TMAEM to its adsorption on the silica films was assessed from the adsorption of AChl, a model compound for the side chain of *p*TMAEM (structure illustrated in Figure 4). As shown in Figure 10, the IR spectrum of AChl at pH 6 (spectrum B, dashed line) had three distinct frequency regions of broad absorbance at 905–989, 990–1117, and 1190–1321 cm^–1^. Previous experimental and computational studies^43–45^ of AChl’s IR spectrum have assigned absorbances in these regions to fundamental and combination vibrational modes of the C–O, C–C, and C–N functional groups of AChl. The difference spectrum for adsorbed AChl on the SiOH_high_ film (Figure 10, spectrum C, solid line) exhibited only a weak absorbance in the 990–1117 cm^–1^ region of the TO mode of the film that peaked at 1087 cm^–1^. The complementary spectra of AChl on the SiOH_low_ film produced a nearly identical difference spectrum as shown for AChl on the SiOH_high_ film in Figure 10. These results suggested that AChl had adsorption comparable to those of both films. However, absorbance peaks in the difference spectrum attributable to distinct peaks of the C–O, C–C, and C–N functional groups of AChl in the adsorption of AChl on the films were not apparent, suggesting that its adsorption on the films is attributable mainly to its quaternary ammonium ion.

**Figure 10:**
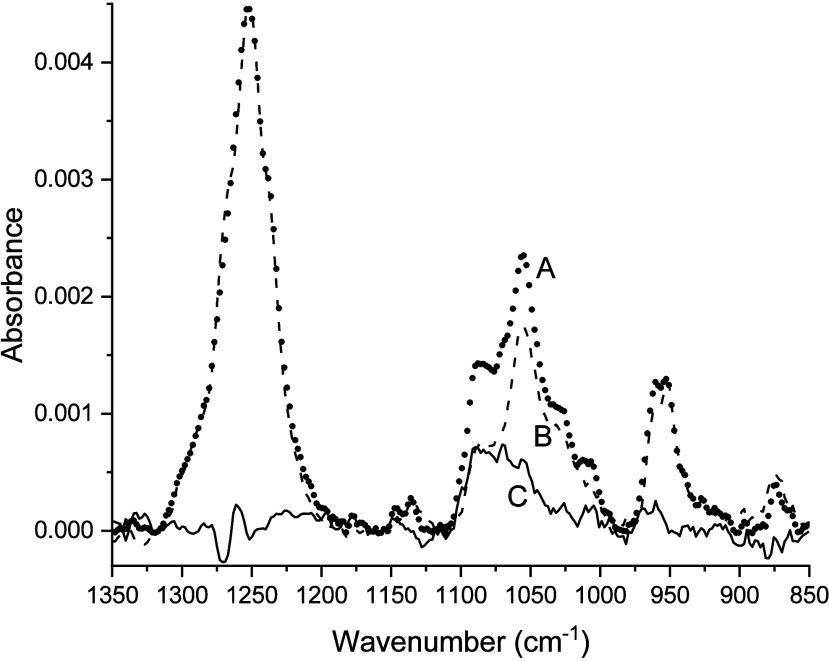
Overlay of IR spectra of pH 6 solutions of 100 mM AChl with a SiOH_high_ film (spectrum A, filled circles), 100 mM AChl with no film (spectrum B, dashed line), and the baseline-corrected, scaled difference spectrum (spectrum C, filled circles = solid line – dashed line).

Unlike *p*TMAEM adsorbed on either SiOH_low_ or SiOH_high_, a pH 6 solution of *p*TMAEM contacting a thick ceria film produced an absorbance peak at 950 cm^–1^, and a broad envelope of overlapping peaks between 1000 and 1200 cm^–1^ peaked at 1109 and 1143 cm^–1^ as shown in Figure 11. These absorbances likely correspond to a mixture of adsorbed *p*TMAEM and nonadsorbed *p*TMAEM as suggested by comparison with the 905–989 and 990–1117 cm^–1^ absorbance regions of *p*TMAEM (spectrum A in Figures 8 and 9). This finding suggested that the C–O, C–C, and C–N groups of *p*TMAEM side chains may produce adsorption on the underlying ceria film supporting the deposited silica film. However, given the use of a thin film (ca. 0.23 μm) of CeO_2_ particles to stabilize the overlaid thick silica films (ca. 1.5 μm), the IR absorbance in the 985–1125 cm^–1^ region for adsorbed *p*TMAEM on a ceria-supported silica film is dominated by the silica layer.

**Figure 11:**
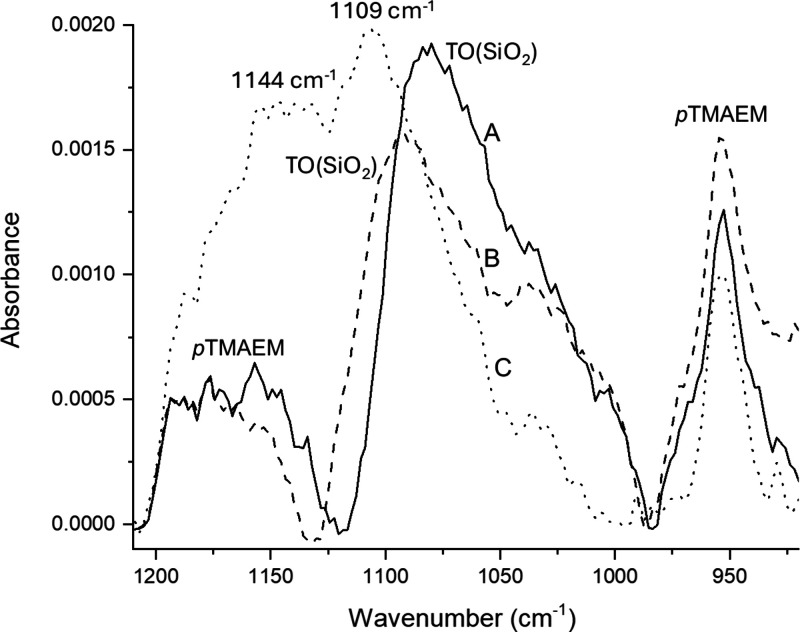
Overlay of IR spectra for *p*TMAEM in pH 6 mixtures with 5 mM *p*TMAEM in the presence of a SiOH_high_ film (solid line), a SiOH_low_ film (dashed line), and a thick CeO_2_ film (dotted line).

### Determination of Langmuir Adsorption Isotherms

The distinctive common feature among the IR spectra of adsorbed TMA, AChl, and *p*TMAEM ions on the silica films was the broad absorbance between 985 and 1125 cm^–1^ due to the phonon modes of the silica films. Representative linearized Langmuir isotherms determined for TMA adsorption on SiOH_high_ and SiOH_low_ films calculated with the peak absorbances of the TO­(SiO_2_) modes of the films are presented in Figure 12. Reliable Langmuir isotherms of adsorbed AChl could not be determined due to the weak absorbance for the TO­(SiO_2_) modes of the films for all concentrations of AChl in equilibrium with the films.

**Figure 12:**
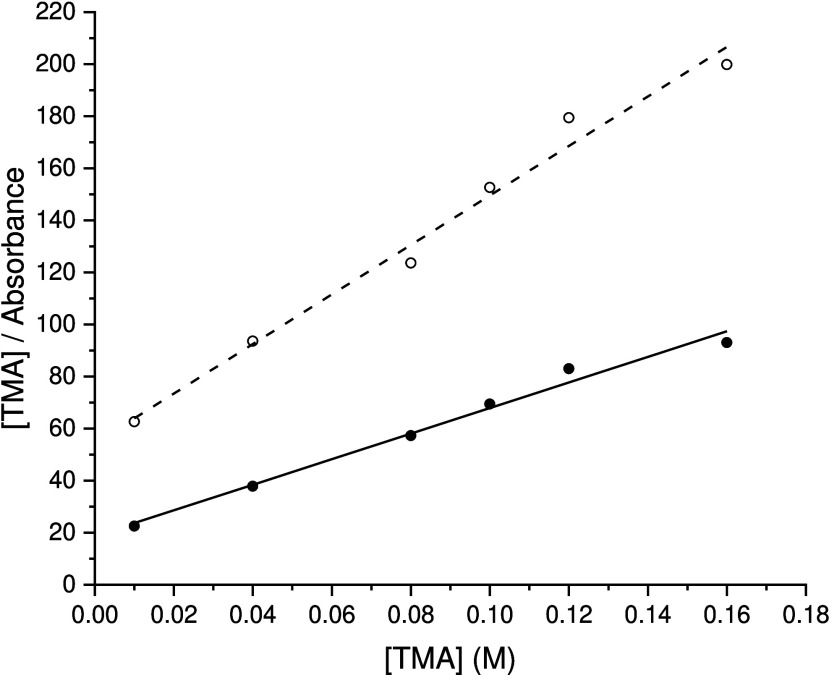
Langmuir adsorption isotherms of pH 6 solutions of TMA on the silica films. Empty circles are experimental points for the adsorption of TMA on the SiOH_high_ film. Filled circles are experimental data points for the TMA adsorption of the SiOH_low_ film. Linear regression fittings to the respective isotherm data points are displayed as dashed (SiOH_high_) and solid (SiOH_low_) lines. The absorbance maxima at 1079 and 1097 cm^–1^ were used for the isotherm determinations with the SiOH_high_ and SiOH_low_ films, respectively.

To directly compare the isotherms for *p*TMAEM and TMA adsorption on a per repeating unit basis, the number-average molar concentration of TMAEM repeating units based on the polycation’s previously reported^46^ number-average molecular weight of 8132 g/mol was used as the concentration in the isotherm analysis for *p*TMAEM adsorption shown in Figure 13.

**Figure 13:**
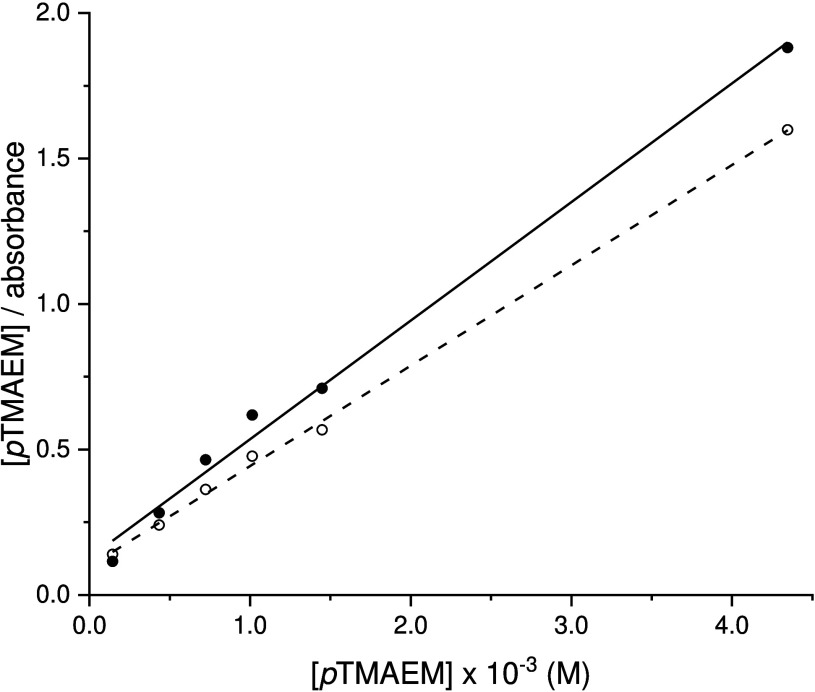
Langmuir adsorption isotherms for pH 6 solutions of *p*TMAEM with the SiOH_high_ film (empty circles) and the SiOH_low_ film (filled circles). Linear regression fittings to the respective isotherm data points are given by the solid (SiOH_low_) and dashed (SiOH_high_) lines. Difference spectrum absorbance maxima at 1089 and 1097 cm^–1^ were used for the isotherm determinations with the SiOH_high_ and the SiOH_low_ films, respectively.

Unlike the isotherm analyses for TMA adsorption, the IR absorbance between 985 and 1120 cm^–1^ for adsorbed *p*TMAEM was obscured partially by C–C, C–O, and C–N absorbances as discussed for the *p*TMAEM spectra in Figures 8 and 9. Using lower *p*TMAEM concentrations, the corresponding absorbances of the silica’s primarily TO phonon band obtained from the baseline-corrected, scaled difference spectra (as shown in Figures 8 and 9) provided the data employed in the *p*TMAEM adsorption isotherm determinations. For *p*TMAEM concentrations exceeding 5 mM, increased silica phonon absorbance was noted and nonlinearity was produced in the isotherms. Based on previously reported studies of polyelectrolyte adsorption on colloidal particles of opposite charge,^9^ multiple-layer adsorption of *p*TMAEM was suggested due to neutralization of the particle’s surface charge at concentrations exceeding a saturating concentration of approximately 5 mM.

Differences between the adsorption of TMA and *p*TMAEM on the silica films are apparent in the isotherms shown in Figures 12 and 13 and in the calculated Langmuir adsorption constants, *K*, summarized in Table 1. Isotherm determinations for TMA and *p*TMAEM adsorption on a thick ceria film, used to model the adsorption of TMA and *p*TMAEM, respectively, on the underlying ceria layer in the ceria-supported silica films, differed significantly from the isotherms for adsorption on the ceria-supported silica films. Based on the *K* values in Table 1, no measurable TMA adsorption and only weak adsorption of *p*TMAEM were found on thick ceria films. These findings support the discussion of the IR spectra of *p*TMAEM adsorption on silica and ceria films in Figure 11 indicating that the silica layer on the films was the primary source of adsorption of TMA and *p*TMAEM.

**Table 1:** Langmuir Adsorption Constants

adsorbed ion	*K* (M^–1^)
TMA on SiOH_high_	17.5 ± 2.2
TMA on SiOH_low_	44.4 ± 11.5
TMA on CeO_2_	^a^
*p*TMAEM on SiOH_high_	3539 ± 480
*p*TMAEM on SiOH_low_	3203 ± 847
*p*TMAEM on CeO_2_	74 ± 28

a Adsorption on film not detected.

The magnitude of the *K* values in Table 1 suggested weaker adsorption of TMA compared to *p*TMAEM adsorption on the SiOH_high_ and SiOH_low_ films. Stronger adsorption of *p*TMAEM was consistent with the multiple adsorptive electrostatic interactions with the silica films resulting from the presence of a quaternary ammonium ion on every TMAEM repeating unit. Increased adsorption of *p*TMAEM was also expected based on previous studies of polyelectrolyte adsorption to charged surfaces.^9^ Previous investigations have established that the interaction of polyelectrolytes with an oppositely charged surface can produce strong adsorption leading to irreversible^47,48^ and reversible^49,50^ adsorption.

Comparison of *K* values for *p*TMAEM adsorption on the SiOH_high_ and SiOH_low_ films indicated that the difference between the values was not statistically significant at the 95% confidence level based on the relative magnitudes of their uncertainties. By contrast, the difference between the *K* values for TMA adsorption on the SiOH_high_ and SiOH_low_ films was statistically significant. The higher value of *K* for TMA adsorption on the less negatively charged SiOH_low_ film suggested that nonelectrostatic interactions may also contribute to the adsorption of TMA on the films. Potential sources of nonelectrostatic interactions are suggested by compositional differences between the films. The SiOH_low_ film will contain a higher proportion of uncharged siloxane groups (Si–O–Si) relative to its silanoate anions (Si–O^–^) compared to that of the SiOH_high_ film. Nonelectrostatic interactions between TMA’s methyl groups and the SiOH_low_ film’s Si–O–Si functional groups are one plausible means to produce differing adsorptive interactions of TMA with the films, thereby altering its Langmuir adsorption constant. Further studies with additional quaternary amines of differing chemical compositions may provide deeper insight into the relative importance of electrostatic and nonelectrostatic interactions on the adsorptive behaviors of colloidal silica particle films.

## Conclusions

The adsorption of quaternary ammonium ions, including TMA, AChl, and *p*TMAEM, onto colloidal silica particle films under aqueous conditions was successfully characterized using a combination of ATR/FT-IR spectroscopy and DFT calculations. Historical challenges with collecting experimental IR spectra for silica films in aqueous solution were effectively resolved by first depositing a thin film of colloidal ceria particles on the ATR’s diamond IRE to stabilize the main colloidal silica particle film. For the TMA–silica complexes, these methods enabled the acquisition of both experimental and calculated IR spectra, visualization and characterization of vibrational modes, and quantitation of Langmuir adsorption constants. The results suggest that both electrostatic and nonelectrostatic interactions influence the adsorption of TMA to silica, while strong electrostatic interactions, likely from the repeating polymeric units, dominate *p*TMAEM’s adsorption to silica. In comparison, AChl’s adsorption to silica was relatively weak with the IR spectra, suggesting that the quaternary ammonium ion was the primary driver of any measurable interaction. The ceria-supported silica film methodology and characterization of quaternary ammonium ion adsorption on silica can be applied to study the adsorption of other molecular ions to better understand and optimize technologies using other materials.

## Supplementary Material



## Notes

Dependence of the absolute value of the integrated IR intensity for the SiOH_low_ film on the solution pH between pH 2 and 10 (Figure S1) and comparison of experimental and calculated IR frequencies of TMA–silica complexes (Table S1) (PDF)
