# Supplementary material for: Spectroscopic and Computational Characterization of the Adsorption of Quaternary Ammonium Ions on Ceria-Supported Colloidal Silica Particle Films
Source: Langmuir. 2025 Aug 13;41(33):22048–58. doi: 10.1021/acs.langmuir.5c01955 (PMC12392718; doi:10.1021/acs.langmuir.5c01955)
Supplement: Supplementary file 1 [file la5c01955_si_001.pdf]

Supporting Information for:

Spectroscopic and Computational Characterization of the Adsorption of Quaternary Ammonium Ions on Ceria-Supported Colloidal Silica Particle Films

Nicole M. Smiddy, Jamie L. Sedlacko, Charles M. Ramia, Patrick E. Schneider,

Dean J. Campbell, Wayne B. Bosma* and Edward E. Remsen*

Mund-Lagowski Department of Chemistry and Biochemistry

Bradley University

1501 West Bradley Avenue

Peoria, IL 61625, USA

Submitted to: *Langmuir*

*Corresponding Authors: eremsen@bradley.edu, bosma@bradley.edu

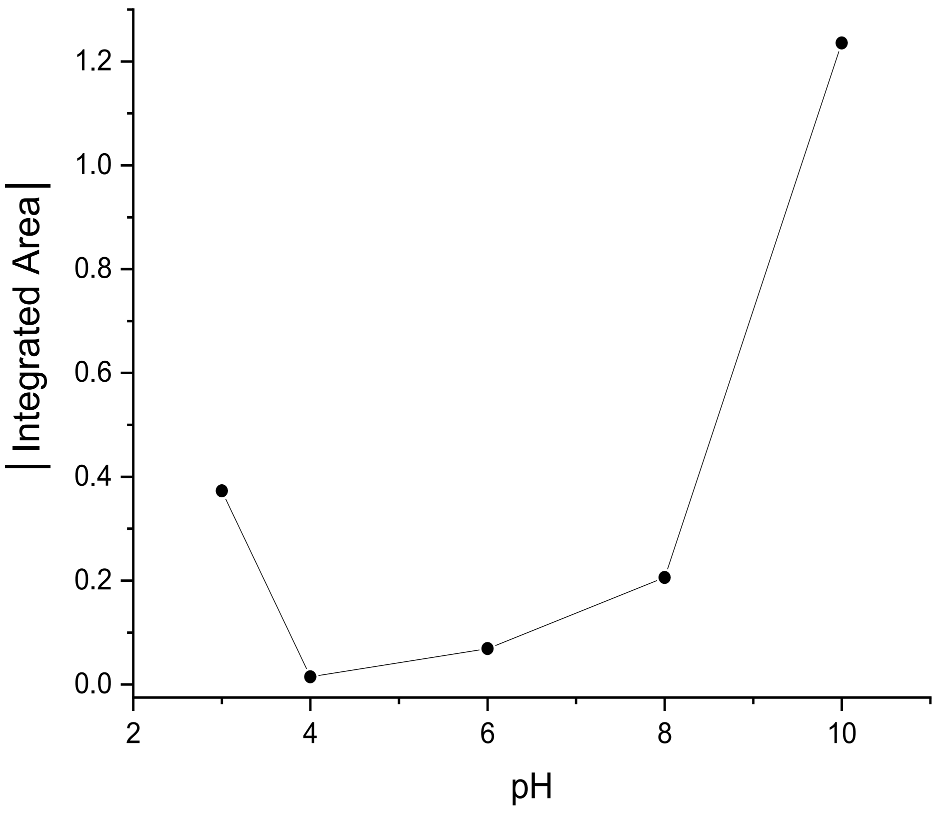

Figure S1. Absolute value of the integrated area of the band corresponding to the 1101 cm^{-1} IR absorbance minimum in the ATR/FT-IR spectra for ceria-supported silica films prepared with $SiOH_{low}$ after 30 minutes of exposure to flowing water with pH ranging from 3 to 10.

Table S1. Infrared peak frequencies for the DFT calculated IR spectrum and the experimental spectrum of 40 mM TMA with a ceria-supported $SiOH_{high}$ film.

Experimental infrared peak (cm^{-1})	Corresponding peak(s) in DFT calculation (cm^{-1})
1489	1548
1162	1187, 1133, 1115
1079	1068
1038	1026
951	958
